# Plasma pharmacokinetic profile and efficacy of meloxicam administered subcutaneously and intramuscularly to sheep

**DOI:** 10.1371/journal.pone.0215842

**Published:** 2019-04-24

**Authors:** Alyssa N. Woodland, Dominique Van der Saag, Benjamin Kimble, Peter J. White, Merran Govendir, Sabrina Lomax

**Affiliations:** 1 School of Life and Environmental Sciences, Faculty of Science, The University of Sydney, Sydney, Australia; 2 Sydney School of Veterinary Science, Faculty of Science, The University of Sydney, Sydney, Australia; University of Bari, ITALY

## Abstract

Plasma pharmacokinetic profiles and the anti-inflammatory efficacy of meloxicam were determined when administered subcutaneously (SC) or intramuscularly (IM) to sheep. Merino ewes were initially injected with 0.1 mL of oil of turpentine into a forelimb to induce inflammation, followed by either 1.0 mg/kg or 2.0 mg/kg of meloxicam administered either SC or IM (n = 6 per treatment group) or followed by no meloxicam administration (control) (n = 4). Ewes were examined to determine skin temperature, limb circumference, limb sensitivity and signs of lameness at 0, 0.5, 1, 2, 4, 6, 8, 10, 12, 24 and 48 h following treatment, with blood collected at these time-points to quantify meloxicam plasma concentrations. Skin temperature of ewes dosed with meloxicam at 1.0 mg/kg SC and 2.0 mg/kg IM at 12 h and 1.0 mg/kg SC at 24 were significantly different to the controls (*P* < 0.05). Limb circumferences of ewes dosed with 1.0 mg/kg IM were significantly different to controls at 10 h and 12 h (*P* < 0.05). All meloxicam treatment groups resulted in reduced limb sensitivity compared to controls at 6 h, with the 1.0 and 2.0 mg/kg IM treatments significantly different at 12 h (*P* < 0.05) and 1.0 and 2.0 mg/kg SC groups, significantly different to controls at 48 h (*P* < 0.05). No significant difference in lameness scores were detected over 48 h. The 1.0 mg/kg IM treatment had a significantly greater plasma meloxicam concentration than the 1.0 mg/kg SC treatment over 0.5 to 4 h (*P* < 0.001). Both 1.0 mg/kg SC and IM treatments demonstrated elimination half-lives (mean ± SD) of 10.82 ± 2.46 and 12.63 ± 2.37 h, respectively. Meloxicam at all doses provided some anti-inflammatory and analgesic effects from 6 to 48 h; however no route could be distinguished as more efficacious than the others.

## Introduction

Meloxicam is a non-steroidal anti-inflammatory drug (NSAID) registered for a number of species including humans, cats, dogs, cattle, pigs and most recently sheep [1]. Mechanistically, meloxicam inhibits the enzyme cyclo-oxygenase (COX) that converts arachidonic acid to prostaglandins [2]. Therefore NSAIDs, such as meloxicam, reduce formation of those prostaglandins (PG), such as PGE_2_, that induce inflammation, pain and fever [3]. Meloxicam is considered to selectively inhibit the COX-2 isoenzyme in humans [4], resulting in low ulcerogenic potential of the gastric mucosa, however this may not be the case for all species [2]. Aspects of meloxicam’s pharmacokinetic profile has been documented for numerous species including dogs [3], goats [5], horses [6], cattle [7] and other domesticated and non-domesticated species [8]. Consequently, meloxicam has demonstrated favourable characteristics in many species, such as an extended elimination half-life of 24 h in dogs [3], 20 h in humans [3] and 8.5 h in horses [9], and a high oral availability such as 106% in dogs [3], 89% in humans [10] and 85% in horses [9].

The plasma pharmacokinetic profile of meloxicam in sheep has been reported for intravenous (IV) administration at 1.0 mg/kg [11] or 0.5 mg/kg [12] and oral administration at 1.0 mg/kg [12]. In addition, subcutaneous injection (SC) of oil of turpentine at 0.1 mg/kg into one front limb to induce inflammation followed by administration of meloxicam at 1.0 mg/kg intravenous injection (IV) in sheep, demonstrated that meloxicam had some effect on reducing inflammation and pain in the treated limb, compared to the turpentine injected limbs of control ewes [13]. Furthermore, meloxicam has been shown to significantly reduce abnormal behaviours associated with pain during husbandry procedures such as castration and tail docking in sheep when administered to the buccal mucosa at 1.0 mg/kg [14]. A description of the pharmacokinetic profile with the efficacy of the registered dose in sheep of 1.0 mg/kg SC is lacking. With known benefits such as on-farm practicality and slower absorption, resulting in potential for longer-lasting analgesia [15], SC administration is thought to be ideal. However it is possible that alternative routes of delivery, such as the intramuscular (IM) route, could be more efficacious for providing pain relief. Other studies investigating NSAIDs in pigs, sheep and horses indicate greater bioavailability and maximum concentrations with IM routes of delivery [16–18].

The objective of this study was to determine the plasma pharmacokinetic profiles and efficacy of meloxicam at the recommended SC dose of 1.0 mg/kg and at a higher dose of 2.0 mg/kg and to compare both 1.0 and 2.0 mg/kg administered IM. Efficacy of meloxicam was assessed by evaluating some physiological and behavioural responses associated with limbs treated with oil of turpentine.

## Material and methods

### Animals and housing

This investigation was conducted at a University of Sydney farm located in Camden, New South Wales (NSW). The sheep used for this current study were randomly selected from the university’s flock maintained for research purposes. This study was approved by The University of Sydney Animal Ethics Committee (Approval 2015/944). Fourteen Merino ewes (*Ovis aries*) (44 to 68 kg; mean 56.8 kg) were housed in a group pen (10 x 10 m) underneath a covered shed with outdoor access. The sheep were acclimatised to housing for two weeks prior to the investigation. During this time, sheep were drenched for internal parasites with Q-drench (Jurox, Rutherford, NSW) at the manufacturer’s recommended dose. One week prior to experimentation, sheep were habituated daily to handling which included being caught, tipped and restrained in lateral recumbency. Sheep also underwent jugular venepuncture intermittently during this period. Sheep were fed an allocation of 750 g/d lucerne hay cubes (MultiCube, Yarrawonga, Vic.). During the 10-day washout period between meloxicam treatments, sheep were released onto a paddock with *ad libitum* access to kikuyu pasture and water. On study completion, the animals were returned to their original flock.

### Treatments

A randomised crossover design with a 10 day washout period was used, the latter being considered adequate for meloxicam elimination in sheep [12]. Inflammation was induced by injection of oil of turpentine (0.1 mL) (Sigma-Aldrich, Castle Hill, NSW) via an 18-gauge needle into the subcutaneous tissue of the pastern (midway between the fetlock and coronet) on a single forelimb of each sheep as previously described [13]. The front limb chosen for turpentine injection was randomised across sheep. Immediately after this injection, sheep were randomly assigned to one of five treatment groups: meloxicam (Metacam 20; Boehringer Ingelheim, Macquarie Park, NSW) administered as a single SC injection at a dose of 1.0 mg/kg (n = 3) or 2.0 mg/kg (n = 3); or meloxicam administered as a single IM injection at a dose of 1.0 mg/kg (n = 3) or 2.0 mg/kg (n = 3); or no meloxicam administered (control) (n = 2). After the washout period, the animals were randomly reassigned to a different treatment group to ultimately result in six animals in each meloxicam treatment group and four animals in the control treatment group.

For all animals treated with meloxicam, blood samples were collected for quantification of meloxicam plasma concentrations. Sheep were restrained in an upright position and blood samples (3 to 4 mL) were collected into lithium heparin vacutainers via jugular venepuncture at 0, 0.5, 1, 2, 4, 6, 8, 10, 12, 24 and 48 hours post drug administration. Samples were immediately centrifuged for 10 minutes at 3500 x g. Plasma was transferred to sterile vials and frozen at—70 °C until meloxicam quantification, which took place within 35 days of collection.

### Physiological and behavioural responses

Physiological and behavioural responses observed in response to oil of turpentine and simultaneous meloxicam administration included limb temperature, limb circumference, limb sensitivity and gait score to detect signs of lameness. The same investigator (ANW) was responsible for all measurements which were taken at 0, 0.5, 1, 2, 4, 6, 8, 10, 12, 24 and 48 hours post drug administration.

Skin temperature was measured using an infrared non-contact thermometer (Jaycar Electronics, Rydalmere, NSW) with a resolution of 0.1 °C. The laser light on the thermometer was aimed at the cranial surface of both affected and unaffected unshaved carpal joints, in the region of the scaphoid and lunate bones, and held at the recommended distance (300 mm). Ambient temperature was also recorded for each collection time-point (USB Temperature/humidity data-logger XC0424, Jaycar Electronics, Rydalmere, NSW).

Limb circumference was measured, to the nearest millimetre, around the proximal aspect of the carpus using anatomic reference points, on both the affected and control limb using a measuring tape.

Limb sensitivity was measured with a calibrated hand-held pressure algometer (Wagner Pain Test FPIX Digital Algometer model FDIX, Wagner Instruments, Riverside, CT, USA) which has a maximum pressure of 10 kgf (kilograms of force). The device consisted of a 1 cm^2^ blunt rubber tip and was applied, with increasing pressure at a perpendicular angle to the target site, midway between the fetlock and the coronet, on both the affected and unaffected limbs of each ewe. The force required for limb withdrawal was recorded as the mechanical nociceptive threshold (MNT) to the nearest 0.5 kgf. When sheep were unresponsive to the maximum applied force, the force was documented as 10 kgf. The hand-held device was returned to zero after each pressure test.

Sheep were assessed for no lameness vs lameness using a numerical rating scale (NRS) based on behavioural characteristics of gait, with behaviours associated for each rating score defined in Table 1. Scoring occurred once sheep were released back into the group housing pen from the holding pen, in which physiological measurements were taken. The observer (AWN) stood within the group pen at a distance of 1 to 2 metres from the flock, observing voluntary movement in individual sheep for 1 to 3 minutes to obtain a lameness score. The observer was blinded to individual treatments. If individual sheep were out of sight, the observer entered the flight zone in order to stimulate movement within the flock and allow view of the desired individual.

**Table 1:** Numerical rating scale (NRS) used to score behavioural characteristics associated with lameness in sheep injected with oil of turpentine.

Score	Associated behaviour
**1**	Even distribution across all limbs with no abnormality in gait
**2**	Mild favouring of limbs, however all limbs used to walk
**3**	Some limping, however all limbs used when walking; reluctance to place limb on ground
**4**	Severe abnormality of gait demonstrated by limited weight bearing on affected limb, increased lying behaviour and pawing affected limb

### Plasma meloxicam concentration quantification and pharmacokinetic analyses

The concentration of meloxicam in plasma was determined by high pressure liquid chromatography (HPLC). The HPLC system comprised of a Shimadzu CBM-20A module (Kyoto, Japan) equipped with a LC-20AT delivery unit with DGU-20As degassing solvent delivery unit and SIL-20AC auto injector. A reversed phase C18 column (Synergi 4μm MAX-RP 80A, 150 x 4.6mm, Phenomenex, Lane Cove, NSW) was used for separation. The isocratic mobile phase comprised of 50 mM potassium phosphate buffer (pH 2.15) and acetonitrile (55:45, v/v). The mobile phase was run at a flow rate of 1 mL min^-1^ with an oven temperature of 30°C. Meloxicam and the internal standard (IS) detection were detected via an SPD-M20A diode array detector (Kyoto, Japan) at a wavelength of 355 nm and Shimadzu class VP data system (software version 7.4) (Kyoto, Japan) calculated the area under the drug peaks.

Piroxicam, the IS, was dissolved in acetonitrile at a concentration of 3.3 ug/mL, and 400 uL of this solution was added to 200 uL of the plasma samples (2: 1 ratio) for protein precipitation. The mixture was vortexed for 5 seconds and centrifuged at 14000 g to produce a precipitate. The supernatant (20 uL) was injected into the HPLC system. Meloxicam plasma concentration within the samples was defined by obtaining standard curves via the analysis of blank plasma samples which were spiked with meloxicam at 0.05, 0.10, 0.2, 0.4, 0.8, 1.50, 3, 6, 12.5 and 25 μg/mL. The retention times of meloxicam and the IS were observed at 8.5 minutes and 5.5 minutes, respectively.

The standard curve was linear from 0.049 to 25 μg/mL. The lowest limit of quantification (LLOQ) was determined by the formula LLOQ = 10 x σ/S whereby σ refers to the standard deviation and S is the slope of the calibration curves. The LLOQ was determined as 0.10 μg/mL with the acceptance threshold defined as precision less than 15% and accuracy within ± 20% of the nominal concentration across analyses as recommended by the International Conference of Harmonisation [19]. Triplicate samples of meloxicam in plasma at 0.2, 2.0, and 20.0 μg/mL were quantified daily (intra-assay) and across three consecutive days (inter-day) for accuracy and precision. Intra-day and inter-day accuracy, calculated as [(estimated value/nominal value) × 100] were 103.8 ± 5.1% and 103.8 ± 4.3%, respectively. Intra-day and inter-day precision was calculated as coefficient of variation (CV) x [(SD/mean value) x 100] were 1.64 ± 1.1% and 1.64 ± 0.3%.

The pharmacokinetic (PK) profile of each meloxicam administration route was determined using a non-compartmental model. The PK values determined were: maximum plasma concentration (C_max_) and time taken to reach maximum plasma concentration (T_max_), as determined by visual inspection of the data; absorption constant (k_a_), as determined by the method of residuals [20]; elimination constant (k_el_) was calculated as the negative slope during the elimination phase of the natural log of drug concentration versus time. The area under the concentration-time curve (AUC_0-t_) and area under the first moment curve (AUMC_0-t_) were calculated to the last quantifiable concentration using the linear trapezoidal method. The elimination half-life (t_1/2_), apparent clearance (CL/F) (where F is the bioavailability which could not be directly calculated as there was no IV administration), apparent volume of distribution (V/F), mean residence time (MRT) and terminal segments of the AUC_0-∞_ and AUMC_0-∞_ were calculated as follows:

$${\text{t}}_{1/2}=\text{ln}\;2/{\text{k}}_{\text{el}}$$

$$\text{CL}=\text{dose}/{\text{AUC}}_{0-\infty }$$

$$\text{V}/\text{F}=\text{CL}/{\text{k}}_{\text{el}}$$

$$\text{MRT}={\text{AUMC}}_{0-\infty }/{\text{AUC}}_{0-\infty }$$

$${\text{AUC}}_{\text{t}-\infty }={\text{C}}_{\text{last}}/{\text{k}}_{\text{el}},$$

where C_last_ is the last measured plasma concentration.

$${\text{AUC}}_{\text{t}-\infty }=\left[\left({\text{C}}_{\text{last}}\times {\text{t}}_{\text{last}}\right)/{\text{k}}_{\text{el}}\right)]+({\text{C}}_{\text{last}}/{\text{k}}_{\text{el}}{}^{2})$$

where C_last_ is the last measured plasma concentration, and t_last_ is the time of the last measured plasma concentration.

Bioavailability (F) was estimated as follows:

$$\text{F}=\left({\text{AUC}}_{\text{extravascular}}/{\text{AUC}}_{\text{iv}}\right)\times \left({\text{Dose}}_{\text{iv}}/{\text{Dose}}_{\text{ev}}\right),$$

where AUC_ev_ is the median AUC_0-t_ calculated following extravascular administration, and Dose_ev_ is the extravascular dose given to effect the AUC_ev_. The median AUC_iv_ and Dose_iv_ were taken from Stock *et al*., 2013.

### Statistical analyses

Skin temperature, limb circumference and limb sensitivity were analysed in GenStat (VSN International Ltd, 14^th^ Edition 2011) with a restricted maximum likelihood linear mixed model (REML). The fixed effects of the model were treatment x time-point and limb and the random effect of the model was individual sheep. Ambient temperature was statistically significant against skin temperature and was therefore included in the model for skin temperature as a random effect using Spearman’s rank correlation. Limb sensitivity data were converted to binomials: 10 kgf = 1 and < 10 kgf = 0 for statistical analysis, however this transformation did not assist in normalising the dataset. Gait scores were subjected to ordinal logistic regression (OLR) in ASReml 3.0 statistical software (VSN International, Hemel Hempstead UK). The fixed effects of the model were treatment x time-point and the random effect of the model was individual sheep. Data from the OLR analysis are presented as cumulative odds ratios with the statistical probabilities of sheep having gait scores of *Y* = 1, 2, 3, or 4. Pharmacokinetic values were also analysed with a restricted maximum likelihood linear mixed model (REML). Pair-wise comparisons were undertaken in Excel (Microsoft Excel 2016 MSO) for any significant treatment x time-point interactions for the physiological and behavioural responses, using least significant differences (LSDs). LSDs were calculated using the formula: 1.96 x average predicted standard error of differences (SED). Distributions of AUC_0-∞_, k_a_, k_el_, elimination half-life (t_1/2_) and mean residence time (MRT) were checked by Shapiro-Wilks normality tests and then compared across the four meloxicam groups with a one-way ANOVA. Any further testing between two means was undertaken by a Tukey’s multiple comparison test. *P* < 0.05 was considered statistically significant for all analyses.

## Results

### Response to the oil of turpentine injection

Sheep demonstrated an immediate hyper-acute response to the oil of turpentine injection by demonstrating restricted weight bearing on the injected limb as well as lying and pawing behaviour. Agitation was evident with the affected limb being held off the ground or frequently raised, up until 12 hours post-administration, with turpentine treated limbs demonstrating a significantly higher skin temperature (mean ± SD: 24.7 ± 2.36 vs 23.31 ± 2.27 °C; *P* < 0.001) and limb circumference (mean ± SEM, 14.35 ± 0.19 vs 13.90 ± 0.16 cm; *P* < 0.001) to the untreated limb.

### Skin temperature

**Fig 1:**
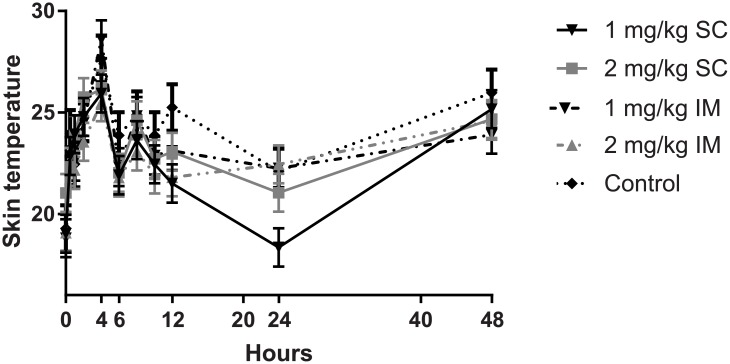
Injected limb skin temperature at selected time-points over 48 h for control group and all four meloxicam treatment groups. There was a significant change in skin temperature between 4 to 6 h (*P* < 0.005). Treatment effects were seen at 12 and 24 h. The 1.0 mg/kg SC and 2.0 mg/kg IM treatments were significantly less than controls at 12 h (*P* < 0.05). At 24 h, the 1.0 mg/kg SC treatment was significantly lower than all other treatments and controls (*P* < 0.05).

There was a significant treatment x time-point interaction (*P* < 0.001) and effect of limb (*P* < 0.001). There was also a significant effect of ambient temperature (*P* < 0.001). As illustrated in Fig 1, there was a significant change in skin temperature of turpentine injected limbs between 4 to 6 h (peak at 4 h, with a decrease at 6 h in all groups) (*P* < 0.005). However treatment effects were seen at 12 and 24 h. The 1.0 mg/kg SC and 2.0 mg/kg IM treatments were significantly less than controls at 12 h (P < 0.05). At 24 h, the 1.0 mg/kg SC treatment resulted in a significantly lower skin temperature than all other treatments, followed by a steady increase in all treatments to 48 h.

### Limb circumference

**Fig 2:**
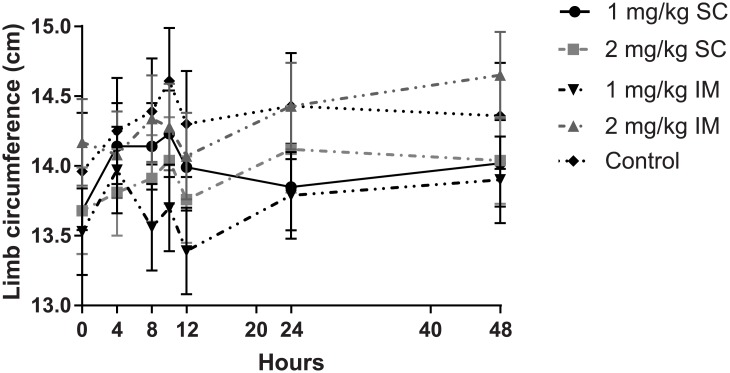
Injected limb circumference at selected time-points for control group and all meloxicam treatment groups. Only the 1.0 mg/kg IM treatment was significantly different to controls at both time-points at 10 and 12 h (P < 0.05).

There was a significant treatment x time-point interaction (*P* = 0.012) and effect of limb on limb circumference (*P* < 0.001). As illustrated in Fig 2, circumference of turpentine injected limbs peaked at 10 h, while the limb circumference of all treatment groups were lower than that of the controls at 10 and 12 h, but only the 1.0 mg/kg IM treatment was significantly different to controls at both time-points (*P* < 0.05).

### Limb sensitivity

**Fig 3:**
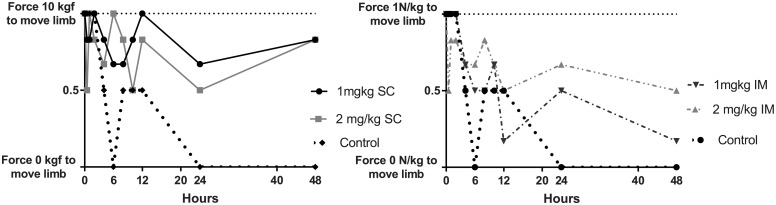
A. Limb sensitivity at selected time-points for control group and 1.0 and 2.0 mg/kg SC treatment groups B. 1.0 and 2.0 mg/kg IM treatment groups. Significant differences between all meloxicam treatments with the control group at 6 h (*P* < 0.05), at 12 h, the 1.0 mg/kg and 2.0 mg/kg IM treatment was significantly different the control group (*P* < 0.05), and at 48 h the 1 and 2 mg/kg SC treatments were significantly different to the control group (*P* < 0.05).

Technical issues with the algometer meant that data on limb sensitivity were collected on the second phase of this study only (n = 3 in each meloxicam group and n = 2 in control group). There was a significant treatment x time-point interaction (*P* < 0.001) and effect of limb (*P* = 0.01). Significant differences between all meloxicam treatments resulted in reduced limb sensitivity as compared to controls at 6 h (*P* < 0.05). At 12 h, the 1.0 mg/kg and 2.0 mg/kg IM treatment were significantly different to controls (*P* < 0.05), and at 48 h, the 1 and 2 mg/kg SC treatments were significantly different to controls (*P* < 0.05) as illustrated in Fig 3, A (SC formulations vs control) and B (IM formulations vs control).

### Gait score

**Fig 4:**
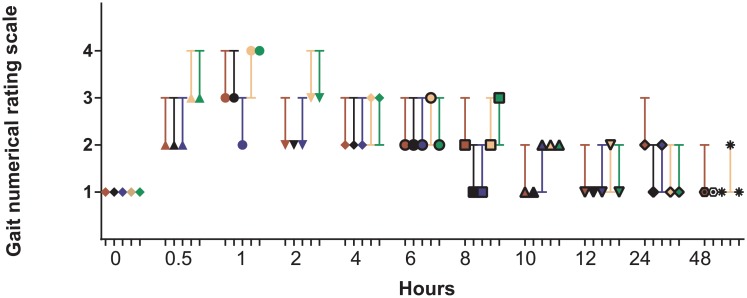
Gait score for controls and all four meloxicam treatment groups. Symbol denotes median, lower bar is 25% quartile; upper bar is 75% quartile. If no upper or lower bar than that missing limit is the same as the median. Symbol and bars: red = control treatment, black = 1 mg/kg SC, blue = 2 mg/kg SC, tan = 1 mg/kg IM, green = 2 mg/kg IM.

There was a significant treatment x time-point interaction (*P* < 0.001). Signs of lameness were most obvious between 0.5 to 2 h with a decrease apparent from 8 to 48 h as illustrated in Fig 4. A lameness score of 2, 3 or 4 was most probable between 0.5 and 8 h, with the 1 h time-point being associated with the highest probability of a lameness score of 4. At 48 h, the 2.0 mg/kg SC treatment was the only treatment observed to return gait of all sheep to a baseline score of 1. Whilst there was a trend for a significant treatment effect, pair-wise comparisons revealed no significant differences between treatments.

### Pharmacokinetic analysis

**Fig 5:**
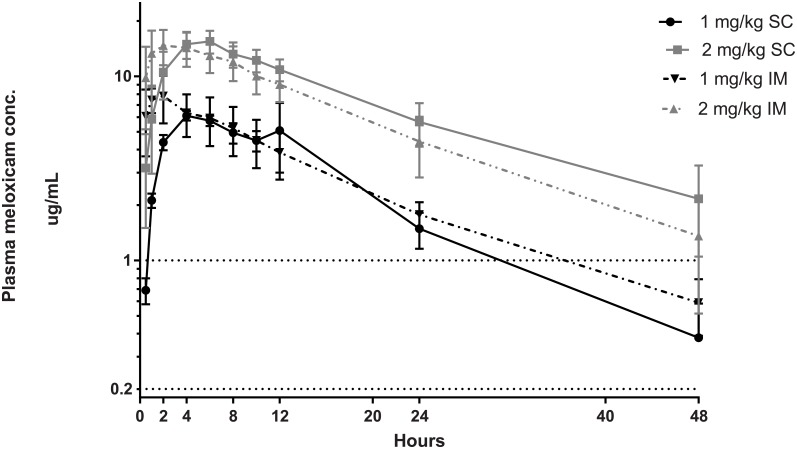
Semi log representation of plasma meloxicam concentration over time of all treatments. Dotted line at 0.2 ug/mL is meloxicam plasma concentration associated with the half maximal effective concentration (EC_50_) that provides pain relief in horses with experimentally induced arthritis [6]. Dotted line at 1 ug/mL approximates effective plasma concentrations to ameliorate inflammation suggested for humans, dogs and horses: 0.13–0.2 [10], 0.82 [21] and 0.57–0.93 μg/mL [22], respectively.

The mean plasma meloxicam concentrations versus time for all four meloxicam treatments are illustrated in Fig 5 and PK indices are summarised in Table 2. There was a significant treatment x time-point interaction (*P* < 0.001). The 1.0 mg/kg IM treatment had a significantly higher meloxicam plasma concentration when compared to the 1.0 mg/kg SC treatment at time-points 0.5, 1, 2 and 4 h. There were no significant differences between any treatments for k_el_, t_1/2_ or MRT however for AUC and k_a_ there were some significant differences documented in Table 2. No adverse effects were observed following 1.0 or 2.0 mg/kg IM or SC administration of meloxicam.

**Table 2:** Pharmacokinetic indices’ mean ± SD for the four meloxicam treatment groups (n = 6 per group).

	SC		IM		Significant P values
	1 mg/kg	2 mg/kg	1 mg/kg	2 mg/kg	
**C_max_ (μg/mL)**	6.96 ± 1.03	15.94 ± 2.25	9.77 ± 2.03	15.30 ± 3.03	
**T_max_ (h)**	4.67 ± 1.03	5.0 ± 1.09	2.08 ± 1.56	2.83 ± 2.79	
**V_z_/F (L/kg)**	0.12 ± 0.01	0.11 ± 0.01	0.11 ± 0.05	0.11 ± 0.02	
**CL/F (L/kg/h)**	0.008 ± 0.002	0.005 ± 0.001	0.006 ± 0.002	0.006 ± 0.002	
AUC_0-t_ (μg/mL*h)	122.19 ± 21.14	313.01 ± 42.63	162.60 ± 40.54	286.29 ± 83.30	
AUC_0-∞_ (μg/mL*h)	129.89 ± 27.54 acd	376.91 ± 77.97 a	174.7 ± 41 bc	320.9 ± 97.78 bd	a: P <0.0001 b: P = 0.0009 c: P < 0.0009 d: P <0.0001
**k_a_(h^-1^)**	0.28 ± 0.11 a	0.44 ± 0.20 b	1.65 ± 0.64 a	1.88 ± 1.18 ab	a: P = 0.005 b: P = 0.01
**k_el_ (h^-1^)**	0.07 ± 0.01	0.05 ± 0.01	0.06 ± 0.01	0.06 ± 0.02	
**t_1/2_ (h)**	10.82 ± 2.46	14.28 ± 2.41	12.63 ± 2.37	12.79 ± 3.49	
AUMC_0-∞_ (ug/mL*h^2^)	2193.70 ± 944.36	8361.82 ± 3114.05	2991.18 ± 691.46	6103.44 ± 3076.31	
**MRT (h)**	16.36 ± 3.34	21.61 ± 3.72	17.36 ± 2.73	18.10 ± 4.40	
**Calculated F using IV data from Stock *et al*., 2013**	1.10	1.54	1.54	1.34	

## Discussion

This is the first study to quantify plasma meloxicam concentrations and provide some observations of efficacy when meloxicam is administered SC and IM to sheep. No treatment group appeared consistently superior to any other. This current study demonstrates that when meloxicam is administered by both SC and IM routes, it provides some anti-inflammatory action and pain relief, but not surprisingly, this occurs later than that observed from 2 to 4 h when meloxicam was injected at 1.0 mg/kg IV [13]. In the current study, statistically significant differences between treatment versus control groups were evident 6 to 48 h after meloxicam administration, and summarised in Table 3. The observations of the current study are similar to another that administered 0.5, 1.0 and 1.5 mg/kg meloxicam SC, 90 minutes prior to the 0.1 mL oil of turpentine injection into the pastern in Merino ewes [23]. Meloxicam, when administered at 1.5 mg/kg SC pre-emptively, did not provide any additional analgesic benefits compared to a 1.0 mg/kg dose and the strongest effects of meloxicam were observed 6 to 9 h after administration [23].

**Table 3:** Summary of treatment effects significant different to controls (P < 0.05).

Treatment	Treatment effects different to control (P < 0.05)
**1.0 mg/kg IM**	i) limb circumference 10 h ii) limb circumference 12 h iii) limb sensitivity 6 h iv) limb sensitivity 12 h
**2.0 mg/kg IM**	i) skin temperature 12 h ii) limb sensitivity 6 h iii) limb sensitivity 12 h
**1.0 mg/kg SC**	i) skin temperature 12 h ii) skin temperature 24 h iii) limb sensitivity 6 h iv) limb sensitivity 48 h
**2.0 mg/kg SC**	i) limb sensitivity 6 h ii) limb sensitivity 48 h

Models to detect meloxicam efficacy in sheep include observing behavioural changes in lambs when meloxicam was injected SC, 45 minutes prior to mulesing [24] and when 0.1 mL of oil of turpentine was injected into a pastern of ewes [13, 23, 25]. Because both the magnitude and type of inflammatory stimulus may be variables that can affect physiological and behavioural responses, it is more informative to compare studies that utilise the same inflammatory stimulus in the same species.

In the oil of turpentine injection model administered to ewes, skin temperature and limb circumference were both used as indicators of inflammation as it was expected that meloxicam would reduce vasodilation and oedema associated with inflamed tissue [13]. In the current study, both were significantly affected by meloxicam administration. The 1.0 mg/kg meloxicam SC treatment resulted in a significant difference in skin temperature as compared to controls at 24 h; and the meloxicam 2.0 mg/kg SC treatment showed a similar trend. In another study when meloxicam was administered at 1.0 mg/kg IV, skin temperature of limbs treated with oil of turpentine was lower than that of the controls from 2 h post-administration [13], with the faster reduction in skin temperature likely attributable to the 100% bioavailability associated with IV administration.

Studies reporting changes in limb circumference are equivocal. Limb circumference of sheep injected with oil of turpentine was reduced from 4 to 24 h following treatment with meloxicam (1.0 mg/kg IV) [13], but steadily increased up to 36 h in sheep administered flunixin, carprofen or ketoprofen orally [25]. This latter observation may reflect a lower NSAID bioavailability via the oral route compared to parenteral routes [26]. The current study found that the 1.0 mg/kg IM meloxicam treatment resulted in a significantly smaller limb circumference as compared to the control group at 12 to 24 h post administration, followed by a steady increase up to 48 h.

Limb pressure sensitivity has previously been effective in assessing the anti-nociceptive action of meloxicam in sheep injected with oil of turpentine [13]. Limb sensitivity increased in sheep receiving no meloxicam for pain relief, and returned to baseline sensitivity by 24 h [13]. The current study showed that the control ewes had increased sensitivity up to 48 h and all meloxicam doses reduced the limb sensitivity compared to the controls from 6 to 12 h via meloxicam IM administration, and 6 to 48 h via SC administration. These results align with previous findings where meloxicam was administered 1.0 mg/kg IV, reduced limb sensitivity between 2 to 10 h [13], and when administered SC, 90 mins prior to the inflammatory stimulus, there was reduced limb sensitivity from 6 to 72 h [23]. It has been reported that differences in apparatus used to detect and quantify limb sensitivity may account for this variation between studies [23]. It was noted in the current study that position of the ewe and the device affected the pressure reading, therefore standardising measuring limb sensitivity is an important consideration for future studies.

An earlier study reports a tendency for lameness scores in sheep treated with meloxicam 1.0 mg/kg IV to be lower at 8 and 24 h as compared to control ewes [13]. When meloxicam was administered SC, 90 minutes prior to the oil of turpentine injection, there was a tendency towards a significant effect of meloxicam treatments reducing lameness at all time-points [23]. In the current study there was a trend for a reduction in gait score, however there were no significant differences between treatments. Lameness has been observed to peak at 24 h post-administration of turpentine in other studies [13, 23], however this current study observed peak lameness between 0.5 to 2 h (NRS scores between 2 to 4) with lameness still observed in some groups at 8 h (scores 1 to 3) as illustrated in Fig 4. At later time-points, all treatments generally demonstrated a higher likelihood towards a low lameness score. Differences in scoring and detecting lameness between the studies, such using video images [13, 23] compared to visual assessment used in the current study, may account for some of the variability in severity and duration of lameness between studies.

As expected, the pharmacokinetic profiles obtained in the current study demonstrate that the IM routes reach peak plasma concentration (T_max_) earlier (approximately at 2 to 3 h) than the SC routes (approximately at 5 h). The 2.0 mg/kg SC dose reached a maximal plasma concentration (C_max_) similar to the 2.0 mg/kg IM dose (15.94 ± 2.25 μg/mL and 15.30 ± 3.03 μg/mL, respectively). There was a significant treatment x time-point interaction (*P* < 0.001) for the meloxicam concentrations within plasma. The IM 1.0 mg/kg treatment had a significantly higher meloxicam plasma concentration when compared to SC 1.0 mg/kg at time-points 0.5, 1, 2 and 4 h. However there was no significant difference between the two treatments at any other time-points. Two studies have previously investigated the pharmacokinetics of IV administration of meloxicam at 0.5 kg/mg in sheep [11, 12]. Meloxicam’s elimination half-life has been reported as 10.85 h [11], and 14.0 h [12], which is consistent with the meloxicam half-lives reported in the current study. An attempt was made to calculate the bioavailability of both routes using published data [12] and these results are provided in Table 2. Although these calculations are not entirely successful because the results exceed 1.00 (complete bioavailability [i.e. F = 1.00] is only expected for the IV route), this may indicate that both the IM and SC routes have high bioavailability and this would be in line with a median bioavailability of F = 0.71 determined for oral meloxicam administration [12].

There are two key observations to consider when interpreting the physiological and behavioural outcomes with respect to the plasma SC and IM, PK profiles. The first observation is that doubling the dosage to 2.0 mg/kg did not generate more obvious efficacious physiological or behavioural responses as compared to the 1.0 mg/kg dosages. It was expected that doubling the dose may result in greater anti-inflammatory action and / or analgesia. There was an obvious increase in C_max_ between the 2.0 and 1.0 mg/kg doses, with no corresponding significant effect on lameness, limb circumference or sensitivity. It is possible that at the higher dose tissue cyclo-oygenase enzymes were saturated and thus there was no further amplitude in physiological and behavioural responses.

The second observation was the significance of the administration route. This aspect addresses the rate of drug absorption. The T_max_ and k_a_ (rate of absorption) were much faster for both IM route doses than for those by the SC route which corresponds to the physiological and behavioural responses occurring significantly earlier for the IM route. There were no statistically significant differences between the mean residence times (MRT) between the IM vs SC for either dose, however the 2.0 mg/kg SC dose had the longest MRT. Although the IM absorption was fastest, the SC route k_a_ was still much faster than the respective rate of elimination (k_el_), suggesting that the drug was rapidly absorbed by both routes which had minimal effect on the drug’s elimination kinetics for each route.

A common method to evaluate the pharmacological activity of NSAIDs is to determine the selectivity of COX inhibition of prostaglandin formation in whole blood *in-vitro* [27]. But *in-vitro* and *ex-vivo* results are often highly variable for some NSAIDs including meloxicam [27]. Therefore it was necessary to undertake clinical trials of the NSAID in the current study to detect physiological and behavioural responses and provide an indication of the clinical efficacy of the NSAID.

The effective plasma concentrations to ameliorate inflammation that are suggested for humans, dogs and horses and humans are 0.13–0.2, 0.82 and 0.57–0.93 μg/mL, respectively [10, 21, 22] however these plasma targets have not been determined for the turpentine oil injection inflammatory stimulus in sheep, nor for any other inflammatory model in sheep. If these ranges are taken as the effective plasma concentration for sheep, then a single administration of any of these dosages should exceed 1 μg/mL for 24 h. Therefore all treatments should result in plasma concentrations greater than the half maximal effective concentration (EC_50_) that provides pain relief in horses with experimentally induced arthritis of 0.2 μg/mL [22] for over 48 h.

Predicting meloxicam efficacy based on plasma concentrations is problematic as NSAIDs are known to be highly bound to plasma proteins (>99%) which can affect their bound to unbound equilibrium [28]. Also, acidic drugs like meloxicam distribute into tissues (at equilibrium 40% of meloxicam mass is in the tissues [10]) and the mass of NSAID in inflammatory exudate can greatly exceed plasma concentrations [29]. A more accurate picture would be to correlate the physiological and behaviour observations with meloxicam’s tissue PK profile or changes in plasma to tissue ratio over time, which should be incorporated into future studies.

One of the risks of increasing the plasma concentration by increasing any NSAID doses is the potential for inducing gastric ulcers and possible other negative effects such as reduced kidney blood flow [2, 29]. However on the basis of a single administration, clinical signs attributable to gastric or kidney dysfunction were not observed in the current study. However, no necropsies were undertaken to examine the gastrointestinal tract or kidney either grossly, or histologically, and this should be considered in future work.

## Conclusions

This study demonstrates that meloxicam when administered at 1.0 or 2.0 mg/kg SC or IM is effective at providing some analgesia after injection of turpentine into the front limbs of sheep. Whilst some observations of anti-inflammatory and analgesic efficacy according to dose and administration route over time were obtained, this study could not significantly distinguish therapeutic efficacy of meloxicam between treatments in sheep. Although the IM route reaches the Tmax faster than the SC route, and the higher dose resulted in greater maximal plasma concentration, there is no clear evidence that the IM route or 2.0 mg/kg SC provided significantly superior analgesia than 1.0 mg/kg SC in Merino sheep. The recommended dosage of 1.0 mg/kg SC or 1.0 mg/kg IM appears to provide significant anti-inflammatory action and analgesia for Merino ewes with some significant differences to untreated controls observed from 6 to 48 h.

## Supporting information

S1 File Data sets of plasma pharmacokinetics of meloxicam when administered at 1.0 and 2.0 mg/kg both SC and IM to sheep; and the physiological and behavioural response data. (XLSX)
